# Biomedical Signal Processing and Modeling Complexity of Living Systems

**DOI:** 10.1155/2012/298634

**Published:** 2012-12-10

**Authors:** Carlo Cattani, Radu Badea, Shengyong Chen, Maria Crisan

**Affiliations:** ^1^Department of Mathematics, University of Salerno, Via Ponte Don Melillo, 84084 Fisciano, Italy; ^2^Department of Clinical Imaging Ultrasound, “Iuliu Hatieganu” University of Medicine and Pharmacy, Cluj-Napoca, Romania; ^3^College of Computer Science and Technology, Zhejiang University of Technology, Hangzhou 310023, China; ^4^Department of Histology, “Iuliu Hatieganu” University of Medicine and Pharmacy, Cluj-Napoca, Romania

Living systems are often maintained by information flows and, as such, they present interesting mathematical problems for example, in the modeling and analysis of spatial structures, self-organization, environmental interaction, behavior, and development. Biomedical signals extract information from the complex phenomena being measured, which are typically a time series having both a regular and random components. Solutions attempt to map general principles, which are used to model how the living systems work. Many researchers have been studying these problems because of their interesting mathematical features and because of their scientific importance. The focus of this special issue is the mathematical analysis and modeling of time series in living systems and biomedical signals. It is mostly interested in the related new development of both theoretical study and practical implementation, either with modeling, complexity, statistics, or signal transformation in living systems.

The papers selected for this special issue represent a good panel in recent challenges. The topics of the research papers and review papers are connected with the living systems and biomedical signals, including modeling dynamical complexity in living systems, for example, network dynamics, mass action, dynamical systems theory, methods for analysis and characterization of dynamical complexity, biomedical signal analysis such as mathematical pattern analysis of biological signals, generative mechanisms of biological signal patterning, implementation of signal analysis algorithms, linking biological structure to biological signal generation, and intracellular signal processing, as well as related models and applications, such as systems theory, biological organization, and biomedical information processing.

This special issue contains 31 papers. In the category of modeling dynamical complexity, L. Sena et al. present a fuzzy model to interpret data of drive performances from patients with sleep deprivation. S. Chen et al. review the modeling of biological intelligence for supply chain management system optimization. M. Li presents approximating ideal filters by systems of fractional order. L. T. Ko et al. present nested quantization index modulation for reversible watermarking and its application to healthcare information management systems. C. Cattani studies on the existence of wavelet symmetries in Archaea DNA. A. Ciancio and C. Cattani present separable transition density in the hybrid model for tumor-immune competition. Q. Guan et al. present solid dynamic models for analysis of stress and strain in human hearts. G. Xiong et al. present theorems and application of local activity of CNN with five state variables and one port. L. Fanea et al. present theoretical compartment modeling of DCE-MRI data based on the transport across physiological barriers in the brain. M. Crisan et al. present a multicriteria optimization model for the study of the efficacy of skin antiaging therapy.

In the category of methods for analysis of dynamical complexity, L. Xu et al. present high resolution remotely sensed small target detection by imitating fly visual perception mechanism. J. Zhang et al. present target contour recovering for tracking people in complex environments. M. Li and W. Zhao present a report on CPNs for asymptotic identity in min-plus algebra. M. Xu and C. Wei present remotely sensed image classification by complex network eigenvalue and connected-degree. J. Wen et al. present a batch rival penalized expectation-maximization algorithm for Gaussian mixture clustering with automatic model selection. M. Štrbac and D. Popovic show a software tool for the prosthetic foot modeling and stiffness optimization. T. Carletti and A. Filisetti present the stochastic evolution of a protocell: the Gillespie algorithm in a dynamically varying volume. 

In the category of biomedical signal analysis, S. Chen and X. Li review the annual progress of functional magnetic resonance imaging for imaging neural activity in the human brain. H. C. Hsin et al. present an adaptive coding pass scanning algorithm for optimal rate control in biomedical images. I. Chiorean et al. present a medicoeconomic index for photo-induced skin cancers. C. Vicas et al. present the influence of expert dependent variability over the performance of noninvasive fibrosis assessment at patients with chronic hepatitis C, by the means of texture analysis. G. Nut et al. present a finite element method applied to a problem of blood flow in vessels. S. Chen et al. summarize recent advances in morphological cell image analysis. C. Yao et al. present motion analysis of live objects by super-resolution fluorescence microscopy. H. Castillejos et al. present wavelet transform fuzzy algorithms for dermoscopic image segmentation. D. A. Mitrea et al. present abdominal tumor characterization and recognition using superior order cooccurrence matrices, based on ultrasound images. Q. Guan et al. present a method for modeling and representation of human hearts for volumetric measurement. X. Li et al. present characteristics of evoked potential multiple EEG recordings in patients with chronic pain by means of parallel factor analysis. A. I. Mitrea et al. present iterative methods for obtaining energy-minimizing parametric snakes, with application to medical imaging. K. Lu et al. present nonlocal means based denoising for medical images. K. T. Q. Dang et al. present detecting epileptic seizure from scalp EEG using Lyapunov spectrum.

Of 67 submissions, 31 papers are selected in this special issue. Of course, the topics and papers are not an exhaustive representation of the area of biomedical signal processing and modeling complexity of living systems. It can be seen that although some solutions and models become available, most problems remain open and research is highly active in this field. In the near future, we expect more contributions that will address all of the key aspects raised above. Nonetheless, the special issue represents the recent concerns in the community and we have the pleasure of sharing them with the readers. 

